# From fitness to flourishing: culturally grounded implementation of the PERMAH model in Philippine physical education

**DOI:** 10.3389/fpsyg.2025.1648656

**Published:** 2025-08-05

**Authors:** Maria Luisa M. Guinto, Lizamarie A. Campoamor-Olegario

**Affiliations:** ^1^College of Human Kinetics, University of the Philippines Diliman, Quezon City, Philippines; ^2^College of Education, University of the Philippines Diliman, Quezon City, Philippines

**Keywords:** good health and wellbeing, Indigenous Psychology, physical education and mental health, positive education, teacher wellbeing

## Abstract

**Background:**

Disruptions in education caused by natural disasters, conflict, or global crises pose significant challenges for educators, particularly in physical education (PE), where embodied learning and social interaction are essential. Following the COVID-19 pandemic, supporting the wellbeing of both students and teachers became a central focus of educational recovery. While the PERMAH model of wellbeing has garnered international attention, this study contributes to the growing body of work by exploring how it may take shape within culturally specific and resource-constrained educational contexts.

**Method:**

This qualitative study examined how Filipino PE teachers implemented the PERMAH model—Positive Emotion, Engagement, Relationships, Meaning, Accomplishment, and Health—to support college students transitioning from online to in-person learning. Two focus group discussions were conducted with 13 instructors from a Philippine public university, all of whom taught parallel PERMAH and non-PERMAH PE classes. Reflexive thematic analysis was used to interpret participant narratives through the lens of Filipino psychology.

**Results:**

Four key themes were generated: (1) *creating meaningful connections*, reflecting teachers' efforts to rebuild rapport and empathy with students; (2) *nurturing safe spaces*, demonstrating how they fostered emotional and physical safety in the classroom; (3) *cultivating enjoyment and engagement*, describe how they promote active participation and enthusiasm; and (4) *enhancing teaching fulfillment*, reveal how they developed their wellbeing and sense of professional purpose while incorporating PERMAH in their classes.

**Conclusion:**

This study presents one of the first culturally grounded applications of PERMAH in a Global South context. The findings suggest that locally adapted wellbeing frameworks, such as PERMAH, may help reframe PE as a more relational and meaningful space, offering insights that can inform curriculum design and educational reform.

## 1 Introduction

Educational disruptions—such as natural disasters, sociopolitical conflicts, or global crises—continue to alter schooling systems. Such events disrupt traditional modes of instruction and compel rapid adaptation from both teachers and students. For students, these shifts often result in reduced engagement, limited access to technology and resources, and heightened stress (Allen et al., 2025; Chatterjee, 2022; Conrad et al., 2022; Davoody et al., 2025; Gillis et al., 2024; Žižanović et al., 2021). On the other hand, teachers face the task of adapting to new teaching methods, keeping students engaged, and enhancing their digital skills. They must also confront the difficulties in planning lessons, creating teaching materials, and assessing student progress online (Haron et al., 2021; Richards and Thompson, 2023; Sánchez-Cruzado et al., 2021; Towers et al., 2025).

Research across disciplines has documented how educators, particularly those in PE, have responded to pandemic-related shifts. Previous studies have highlighted both the resilience and the emotional and pedagogical challenges that PE teachers faced when adapting to online or hybrid learning environments, particularly amid prolonged uncertainty (Centeio et al., 2021; Korcz et al., 2021; Mercier et al., 2021; Varea and González-Calvo, 2021; Varea et al., 2022). These experiences underscore the need for supportive frameworks prioritizing wellbeing and academic resilience.

### 1.1 The importance of wellbeing in education

In the aftermath of the pandemic, the interconnections between mental health, physical activity (PA), and academic success—particularly among college students navigating critical developmental transitions—have become increasingly evident (Jiang and Zhang, 2024; Mieziene et al., 2022; Tape et al., 2021). Wellbeing is now widely acknowledged as a multidimensional construct, encompassing physical, social, emotional, and spiritual dimensions, and is vital for both students and teachers (Banggawan et al., 2024; Norozi, 2023; Strukova and Polivanova, 2023). A growing body of literature emphasizes the importance of culturally responsive approaches to wellbeing that go beyond surface-level happiness to foster purpose, competence, and relational depth (Joshanloo et al., 2021; Steenkamp, 2021). However, despite increased awareness and interest, schools continue to face significant barriers in consistently delivering wellbeing programs, highlighting the need for adaptable, locally grounded approaches to embedding wellbeing into educational practice (Carter and Andersen, 2019; Schachner et al., 2021; Wong et al., 2021).

### 1.2 The case of physical education

The difficulties of returning to in-person classes are especially noticeable in PE, a discipline reliant on hands-on, interactive, and participatory learning. Research has revealed that PE teachers struggle with adapting physical activities to virtual platforms, leading to reduced student participation and a pedagogical identity crisis (Centeio et al., 2021; Mercier et al., 2021; Varea and González-Calvo, 2021; Varea et al., 2022). PE teachers face unique challenges, such as ensuring safety and curriculum quality while translating movement-based activities into digital contexts (Korcz et al., 2021). The resulting absence of PA and social interaction hindered instructional quality and student wellbeing (Fan et al., 2025; Goh, 2021; Mesias and Pelicano, 2024). As schools resumed in-person classes, PE was reconceptualized—not merely as a fitness-oriented discipline but as a vital source for promoting social connection, emotional resilience, and holistic wellbeing (Centeio et al., 2021; Varea et al., 2022). However, how such frameworks are appropriated in culturally and economically diverse settings remains underexplored.

### 1.3 Adaptations of the PERMAH framework in the Philippines

The PERMAH framework—an expansion of Seligman's original PERMA model 2011—provides a structured approach to wellbeing in education through six domains: Positive emotions, Engagement, Relationships, Meaning, Accomplishment, and Health (Kern, 2022). International studies confirm its benefits for student motivation, classroom climate, and teacher wellbeing (Chu, 2022; Dorri Sedeh and Aghaei, 2024; Geier and Morris, 2022).

This study builds on recent efforts to localize PERMAH in the Philippines through Filipino Indigenous Psychology, which emphasizes relational values such as *loób* (relational selfhood or inner self shaped by social harmony), *kapwa* (shared inner self or interconnectedness with others), and *pakiramdam* (empathic sensitivity to others' feelings and unspoken cues) (Pe-Pua and Protacio-Marcelino, 2000; Aguiling-Dalisay, 2013). Campoamor-Olegario et al. (2025) illustrated how the PERMAH can be meaningfully integrated into Philippine PE classes by drawing from cultural values that align closely with PERMAH's emphasis on relational flourishing and offered a model for adapting positive psychology to Global South educational contexts. Their work also showed that integrating positive education principles into PE, when rooted in local culture and social realities, empowered students to lead healthier and more joyful lives. These findings are echoed in Kulandaiammal and Neelakantan (2024) review of 16 studies across different countries, which affirmed that PERMA-based interventions in higher education significantly enhance wellbeing, motivation, engagement, and life satisfaction, while also reducing stress, anxiety, and academic boredom. This growing body of evidence highlights the transformative potential of PERMA across diverse educational contexts.

### 1.4 Distinctive contribution of the study

While the PERMAH framework is widely recognized in the field of positive education, its application within PE pedagogy, particularly in Global South contexts, remains underexplored. This study contributes to that growing area of inquiry by examining how Filipino PE teachers interpreted and implemented PERMAH during the transition from online to in-person learning following the COVID-19 pandemic.

This research makes four key contributions. First, it employs a *comparative, teacher-reported design* in which participants simultaneously taught two sections of the same PE course—one incorporating PERMAH principles and one following a traditional format. This unique setup enabled rich, experience-based comparisons between pedagogical approaches. Second, the study focuses exclusively on teacher observations and reflections. It offers a *dual perspective on wellbeing* encompassing the educators' perceived student outcomes and reflections on their professional and emotional growth. No student data were collected; all findings are based on the teachers' comparative experiences. Third, the research is situated in the Philippine higher education context, contributing regionally grounded insights to the global conversation on post-pandemic education. Fourth, it provides an example of how the PERMAH framework can be culturally adapted within the Philippine context, where communal values, relational teaching, and resource limitations influence everyday educational practice.

By reframing PE as a site for the mutual flourishing of students and teachers, the study extends the application of PERMAH into a domain where its holistic potential has been underutilized. In doing so, it highlights the model's value in promoting student engagement and wellbeing, rather than solely focusing on physical development, and in sustaining teacher motivation, creativity, and fulfillment during transition and recovery.

### 1.5 Grounding the study in context

This study is grounded in the Positive Psychology framework of PERMAH (Seligman, 2011; McKenna, 2019; Norrish et al., 2013), contributing to the growing field of positive education. It explores how the six pillars of flourishing were intentionally integrated into higher education PE instruction in the Philippines. Data collection, coding, and thematic analysis were aligned with the PERMAH dimensions, allowing for a structured yet culturally nuanced understanding of teacher-reported experiences. Importantly, this research is situated within a Philippine public university system, where many educators face resource constraints, large class sizes, and a deep sense of service to the nation in nurturing students holistically.

By incorporating Filipino Indigenous Psychology, the study reframes PE as more than a subject for physical training but a wellbeing space—one that fosters not only physical fitness but also *kapwa* (shared identity), *loób* (inner self), *pakiramdam* (sensitivity to others), and *pagpapakatao* (becoming fully human). These cultural values are not just add-ons; they are integral to how wellbeing is understood, expressed, and cultivated in Filipino classrooms.

In this light, integrating PERMAH into curriculum design is both proactive and preventive, supporting resilience, emotional connection, and collective growth. This culturally grounded adaptation positions PE as a vital site for educational transformation in the Philippines, where flourishing is not an individual accomplishment but a shared experience of reconnection, resilience, and becoming truly human.

Positioned at the intersection of global frameworks and local values, this study inquires how PERMAH was interpreted and lived out by Filipino PE teachers. Their voices provide insight into what it means to teach for wellbeing in a context shaped by disruption, culture, and care.

### 1.6 Research questions

This study aimed to explore the perceptions and experiences of Filipino PE teachers who utilized the PERMAH model to support the holistic wellbeing of college students during the transition from remote to in-person learning following COVID-19 disruptions. Specifically, it answered the following research questions:

1. How do Filipino PE teachers implement the six PERMAH domains in higher education settings during the transition from remote to in-person learning?
2. What differences do teachers observe in student engagement, wellbeing, and learning outcomes between PERMAH-infused and traditional PE classes?
3. How does PERMAH integration affect PE teachers' professional experiences, sense of purpose, and wellbeing?

By capturing the experiences of teachers who actively engaged in pedagogical innovation during recovery and reintegration, the study offers valuable insights into how PE can be reconceptualized as a site for mutual flourishing.

## 2 Methodology

### 2.1 Research design

This study employed a qualitative research design grounded in a constructivist paradigm, specifically thematic analysis of semi-structured focus group discussions. It aimed to explore how Filipino PE teachers in a public university system interpreted and implemented the PERMAH wellbeing model in their teaching upon returning to in-person instruction after the pandemic.

### 2.2 Researchers' positionality

The research team brought diverse yet complementary perspectives to the study. Both authors are Filipino educators from the Global South, bringing their shared cultural lens that influenced their interpretation of the relevance and application of the PERMAH framework in PE. This positionality helped them understand how the teacher-participants' actions reflected relational aspects, community, and recovery. The first author is a sport psychologist with extensive experience in teacher training and psychosocial interventions in educational settings. The second author is an educational psychology expert with rich professional experience in teacher education and socio-emotional learning.

### 2.3 Participants and recruitment

We invited a total of 16 PE instructors from an extensive Philippine public university system to participate in our research program. They were tasked with teaching one of their classes using PERMAH and another equivalent class following the standard physical education curriculum, without intentionally integrating PERMAH's wellbeing components.

The group included individuals with varied teaching backgrounds, course types, genders, and educational attainment, reflecting a maximum variation sampling approach that aimed to capture diverse perspectives and enrich the overall analysis. Of these, 13 instructors (eight female and five male) agreed to participate in the focus group discussion for this study. Their profiles and the types of PE classes they taught are presented in Table 1.

**Table 1:** Teacher-participant demographics and assigned physical education classes in PERMAH and Non-PERMAH sections.

**Participant's pseudonym**	**Sex**	**PE classes taught with two approaches: PERMAH and Non-PERMAH**	**Years of teaching**	**Highest level of education**
Allan	Male	Walking for fitness	5	Master's
Brenda	Female	Philippine folk dance	25	Bachelor's
Carla	Female	Walking for fitness	34	Bachelor's
Dahlia	Female	Line dance	6	Ongoing Master's
Ella	Female	Walking for fitness	19	Bachelor's
Fred	Male	Circuit training	9	Bachelor's
Gabby	Male	PE2 Aikido	16	Ongoing Master's
Hilda	Female	Expressive dance	8	Master's
Ivan	Male	Circuit training	12	Bachelor's
Jason	Male	Line dance	7	Bachelor's
Kristine	Female	Circuit training	15	Master's
Linda	Female	Pilates/Social dance	36	Master's
Mila	Female	Modern jazz	5	Ongoing Master's

### 2.4 Ethical considerations

Ethical approval for this study was obtained from the University of the Philippines Manila Ethics Review Board, under Reference Number RGAO-2022-0760. All participants provided informed consent before participation, and measures were taken to ensure confidentiality and anonymity throughout the research process.

### 2.5 Data collection

Data were gathered through two 90-min semi-structured focus group discussions (FGDs), conducted via the Zoom conferencing platform. Although the number of FGDs was modest, the dataset achieved conceptual saturation, with similar patterns, narratives, and interpretive insights recurring during the second session.

Teachers were invited to reflect on the differences between their two classes, focusing on how the application of PERMAH principles influenced student engagement, wellbeing, and their own teaching experience. To guide the reflective discussion, participants were asked to identify classroom practices they intended to continue, those requiring improvement, and those they considered discontinuing. This approach facilitated intuitive and candid sharing, enabling participants to articulate authentic emotional, relational, and pedagogical dynamics within the classroom. All discussions were recorded and transcribed verbatim for analysis.

### 2.6 Data analysis

Data were analyzed using reflexive thematic analysis, a method well-suited for identifying and interpreting patterns of meaning across qualitative datasets. Guided by Braun and Clarke's framework, the study employed a rigorous, iterative process of immersion in the data, inductive coding, theme development, and refinement through reflexive engagement (Braun and Clarke, 2006, 2019). Initial coding was conducted independently by the two researchers to uphold interpretive credibility, followed by collaborative discussions to refine and define themes. Throughout the process, a coding log and analytic memos were maintained to ensure transparency and establish an audit trail (Nowell et al., 2017). This approach enabled the researchers to uncover the nuanced, relational, cultural, and pedagogical insights that teachers associated with embedding wellbeing in their PE classes.

To establish trustworthiness, the study addressed four foundational criteria: credibility, dependability, transferability, and confirmability (Korstjens and Moser, 2017). Credibility was supported through peer debriefing, detailed transcription, and member checking, where participants reviewed and validated researchers' interpretations. Dependability was strengthened through systematic documentation of analytic decisions and procedures. Transferability was enhanced by providing detailed descriptions of the research context and participants' experiences, enabling readers to assess their relevance to other settings. Confirmability was ensured through reflexive journaling and analytic memos, which helped anchor the findings in the data rather than in the researcher's assumptions.

Reflexivity was woven throughout the analytic process. The research team held regular discussions to examine their positionalities and potential influences on interpretation (Braun and Clarke, 2021). These reflexive dialogues, memo writing, and reflective logs helped foster critical awareness and minimize interpretive bias. As reflexive thematic analysis acknowledges the researchers' active role in meaning-making, sustained reflexivity is essential to ensure that interpretations remain thoughtful, context-sensitive, and grounded in participants' perspectives.

Together, these methodological strategies ensured that the themes were theoretically grounded and authentically representative of participants' lived experiences, aligning with the standards of rigorous qualitative research in positive psychology.

## 3 Results and discussion

This section presents the study's findings alongside their interpretation, following the common qualitative practice of integrating results and discussion. In qualitative research, data and meaning are deeply intertwined; therefore, presenting each theme with corresponding analysis enables a richer and more coherent understanding of the participants' experiences. This approach preserves the authenticity of the participants' voices through direct quotations and situates their insights within relevant cultural, psychological, and theoretical frameworks. Organizing the findings thematically, each section weaves together empirical evidence, interpretive insights, and reflections on Filipino cultural values to explore how the PERMAH framework may support meaningful shifts in PE teaching and learning.

The reflexive thematic analysis yielded four interrelated and meaningful themes illuminating how PE teachers perceived and applied the PERMAH wellbeing model during their transition back to face-to-face instruction. First, the theme of *creating meaningful connections* highlights how teachers recognized the value of PERMAH in rebuilding the relational fabric of the classroom. After extended periods of remote learning, teachers deliberately employed strategies grounded in empathy, positive communication, and emotional attunement to restore student-teacher and peer relationships that had been strained or lost during online instruction. This result aligns with findings of Tagare (2023) that PE teachers had to creatively adjust their strategies during the return to face-to-face learning to rebuild human connection, even while navigating the restrictions of health protocols.

Second, the theme of *nurturing safe spaces* reflects the teachers' efforts to establish environments where students feel physically and emotionally secure. Teachers described how the principles of PERMAH helped them structure classes that prioritized psychological safety and health-conscious practices, enabling students to re-engage with PA and social interaction without fear of judgment or exposure to risk.

The third theme, *cultivating enjoyment and engagement*, highlights the role of PERMAH in rekindling students' interest and participation in PE. Teachers shared how they intentionally incorporated fun, challenge, and personal relevance into their lessons, which fostered renewed enthusiasm and increased students' willingness to engage actively in classroom activities.

Lastly, the theme of *enhancing teaching fulfillment* sheds light on the teachers' reflections regarding their wellbeing and sense of professional purpose. Many reported that implementing PERMAH-infused approaches supported their students' holistic development and contributed to their professional satisfaction and emotional resilience. The reciprocal nature of wellbeing was evident, as teachers described feeling more motivated, valued, and connected to their work.

These four themes collectively offer insight into how PERMAH served as a pedagogical framework and a catalyst for emotional reconnection, engagement, and wellbeing within the transitional context of post-pandemic education. The following sections present each primary theme, along with its corresponding secondary themes, in greater detail. Drawing on the participants' narratives, the nuanced ways in which the PERMAH model was experienced and enacted in their teaching practices are illustrated. Table 2 presents the four primary themes, their corresponding secondary themes, and exemplar data extracts.

**Table 2:** Thematic analysis of teacher narratives on PERMAH-infused physical education classes.

**Key themes**	**Secondary themes**	**Exemplar data extracts**	**PERMAH domains tapped**	**Filipino indigenous values**
Creating meaningful connections	Relational teaching and teacher-student interdependence	“There was a greater level of interdependence, not just within the students but with the teacher.” – Allan “For some reason, in the PERMAH group, it always feels like I don't want the class to end... I always accommodate them.” – Dahlia	relationships, meaning	kapwa, pakikipagpalagayang-loób, pakikiisa
	Peer bonding and social belonging	“I found that my students became closer to each other.” – Jason “They help each other, especially if someone misses a class—they share the choreography and information.” – Hilda	relationships, engagement	pakikisangkot, pakiramdam, pakikipagkapwa
	Agency, autonomy, and safe belonging	“They always have the freedom to choose... They felt free to join different groups.” – Brenda “Many students shuffle between groups... they feel at home in all groups.” – Dahlia	Accomplishment, engagement, relationships	loób, pakiramdam, kapwa
Creating safe spaces	Reframing learning through emotional and psychological safety	“I am competing with myself rather than competing with somebody else, and I'm happy where I am.” – Allan (student quote as recalled by the teacher) “My growth, on my learning, on developing my relationship, and how I can grow with other people.” – Dahlia (student quote as recalled by the teacher)	positive emotion, engagement, health	pakiramdam, pagpapakatao, loób
	Enabling authentic expression	“(The activities) made them feel comfortable… made them feel open to express more of what they feel.” – Brenda “It gave them courage to express unspoken feelings.” – Fred	positive emotion, relationships	pakikipagpalagayang-loób, loób
	Strengthening community through relational bonding	“Normally, the discussions and sharing continue even after my classes.” – Gabby “They continue to bond after classes.” – Gabby	relationships, meaning	kapwa, pakikipagkapwa, pakikiisa
Cultivating enjoyment and engagement	Promoting consistent participation and purposeful goal-setting	“PE allowed them to express their personal goals... they monitored themselves.” – Mila “They're excited to share.” – Carla	engagement, accomplishment. positive emotion	layunin, pakikiisa
	Fostering creativity, fun, and intrinsic motivation	“They're more motivated to engage themselves in the activity.” – Hilda “They are very, very happy with how they perform.” – Allan	positive emotion, engagement	malikhain, katuwaan, ginhawa, loob
	Re-energizing teacher practice and learning culture	“Also fortified was my creativity as a teacher in dealing with the lesson.” – Brenda “I was eager to attend the PERMAH sessions.” – Ella	engagement, meaning	ginhawa, malikhain,
Enhancing teaching fulfillment	Rediscovering joy and purpose in teaching.	“I really enjoyed teaching this PERMAH class... more fulfilling as an instructor.” – Allan “I felt freer while teaching the PERMAH group.” – Hilda	meaning, positive emotion	pagkatao, saysay, ginhawa
	Building compassionate and responsive teacher-student relationships.	“It drew me a lot closer to some of the students who were able to open up to me.” – Brenda	relationships, positive emotion, meaning	paninindigan, pakikipagkapwa
	Teaching rooted in Filipino values and shared humanity	“I felt more human in my PERMAH class.” – Hilda “All the activities and the additional drills were beneficial to everyone, including the instructor and me.” – Allan	meaning, relationships	pagkatao, ginhawa saysay

### 3.1 Creating meaningful connections

This first primary theme encapsulates how the PERMAH framework enabled PE teachers to cultivate deeper, more authentic relationships within their classes, between teacher and student, and among peers. Empathy, mutual care, and collaboration may help foster stronger and more meaningful interpersonal relationships. Campoamor-Olegario et al. (2025) discusses that PE teacher's value building supportive relationships in their classrooms, which highlights how well this aligns with the PERMAH model's focus on positive relationships as a key element of student wellbeing.

At the core of Filipino relationality is the concept of *kapwa*—a shared inner self, which is the root word of *pakikpagkapwa*. It refers to recognizing the other as a co-equal person, affirming interconnectedness, and upholding the dignity and humanity of every person. Santiago (1976, as cited in Enriquez, 1986) describes this as “humanness to its highest level” (p. 12). In the PERMAH-infused learning environment, teaching became a relational, not transactional, process. Students saw each other and their teacher as *kapwa—*companions in a shared learning journey. The meaningful connections formed in these classes humanized the learning experience. They became a defining feature of the PERMAH-infused learning environment, contrasting notably with the more transactional or individualistic classroom dynamics often experienced in non-PERMAH class settings. This finding mirrors Candeias et al. research (2024), showing that when educators communicate with empathy and openly share experiences, it helps build a sense of wellbeing and creates a more supportive educational community.

#### 3.1.1 Relational teaching and teacher-student interdependence

Another integral concept in Filipino relationality is *loób*, often translated as “inner self” or “inner being.” It encompasses a person's thoughts, feelings, and intentions—an essential aspect of how one relates to others. In Filipino culture, building trust and meaningful relationships involves a gradual and respectful process of easing one's *loób* to another's, a dynamic known as *pakikipaglagayang-loób*. This process refers to the development of mutual trust, understanding, and rapport—an attunement of inner selves.

Teachers reported that fostering relational depth was central to their pedagogical approach under the PERMAH-infused classes. This depth was not incidental but intentionally cultivated through personal connections. Jason shared how adding a personal touch to his interactions with his students made a difference. “I tried to memorize their names, their courses, and talk about it in class.” Allan similarly reflected that while course content and requirements remained constant across classes, “there was a greater level of interdependence, not just within the students but with the teacher.” Such intentional efforts created a classroom culture marked by relational interdependence, where teaching became attuned not just to learning outcomes but to the lived inner worlds of students. This aligns with findings from other research, which emphasize that empathetic communication and shared experiences are crucial for building supportive relationships and enhancing wellbeing in educational settings (Campoamor-Olegario et al., 2025; Candeias et al., 2024).

This relational dynamic gave rise to enhanced student engagement, or *pakikisangkot* (getting involved)—the second level of Filipino social interaction (Pe-Pua and Protacio-Marcelino, 2000). Dahlia described this deep connection: “For some reason, in the PERMAH group, it always feels like I don't want the class to end... I always accommodate them when they say, ‘Ma'am, I want to stay in class to practice.”' Her willingness to extend time and space for continued engagement reflects both student enthusiasm and the teacher's emotional investment. Allan echoed this sentiment, describing how the PERMAH class, while requiring more effort, was “a lot more fulfilling” due to the strong interpersonal engagement that developed. This willingness to go beyond formal instruction mirrors Nakamura and Csikszentmihalyi (2014) concept of flow, “characterized by complete absorption in what one does” (p. 89).

Eventually, both teachers and students entered a relational depth characterized by *pakikiisa—*being one with the other—the highest form of Filipino social interaction. *Pakikiisa* reflects oneness, solidarity, and a complete display of trust among *kapwa* or shared selves. This culmination of relational teaching practices aligns with global movements toward relational pedagogy (Su and Wood, 2023) and culturally respectful teaching approaches that prioritize care, inclusivity, and co-flourishing (Dietz and Rhodes, 2019). In this view, the Filipino PE class emerges not merely as a site of physical activity and fitness but as a relational space—one where both students and teachers thrive through mutual recognition, shared humanity, and deep interpersonal connection.

#### 3.1.2 Peer bonding and social belonging

The PERMAH-infused learning environment also demonstrated changes in peer dynamics, fostering a classroom marked by closeness, support, and collaboration. This sense of closeness was essential after pandemic-related classroom adjustments, which had limited student interaction and disrupted peer relationships. PERMAH strategies helped restore motivation and rebuild a supportive learning environment where collaboration could thrive (Tagare, 2023).

Hilda observed that students in the PERMAH class were “closer, and they feel more confident in expressing themselves through dance,” noting that movement and expression became pathways for building friendships. This sense of connection extended beyond structured class hours. Gabby shared, “More exchanges are happening in my PERMAH class compared to my non-PERMAH class; they even continue to bond after classes.”

Students took the initiative to support one another in both formal and informal ways. Hilda noted, “They help each other, especially if someone misses a class—they share the choreography and information.” Linda added that students even “created a group chat where they checked the effect of the exercises so that their classmates could see and share them.” These efforts reflect a learning environment where cooperation replaces competition, and peer accountability enhances participation. Studies show that PERMAH strategies, making thoughtful changes in the classroom environment, and responding to student demotivation or misbehavior are key to rebuilding a sense of social belonging (Campoamor-Olegario et al., 2025; Candeias et al., 2024). Silverman and Mee (2019) further reinforce this insight by showing how community circles in schools create safe, intentional spaces where students can openly share their emotions, build trust, and strengthen a sense of mutual support and belonging.

Teachers also recounted moments where students celebrated each other's progress. Kristine reflected, “They were prouder that their classmates learned swimming... especially if they feared water... the class is very happy when they all learn to swim and overcome their fears.” This climate of empathy and collective pride shows how accomplishment was shared and celebrated collectively rather than individually.

Such relational behaviors further illustrate the Filipino values of *pakikisangkot* and *pakikipagkapwa*. These values shaped a strong foundation of belonging in the classroom community, where emotional support, mutual care, and shared joy created a space for students not only to learn but also to flourish together.

#### 3.1.3 Agency, autonomy, and safe belonging

The infusion of PERMAH into PE classes fostered not only interpersonal connection but also student agency and group autonomy—key elements in building safe spaces for belonging. Teachers recalled intentionally giving students the freedom to form their groups or move to another group, creating space for relational ease and choice. Brenda explained, “They always have the freedom to choose...They felt free to join different groups...Whether they stay in that group or move to another group with the next task.” By allowing students this flexibility, teachers promoted what Rodriguez (2023) describes as *collective autonomy*—the culturally grounded source of Filipino agency. Research also reveals that supporting student autonomy enhances vitality and psychological wellbeing, underscoring the importance of offering choice in the classroom (Liu et al., 2017).

This autonomy had visible side effects. Dahlia remarked, “Many students shuffle between groups... It seems that they are not shy to shift groups as if they feel at home in all groups.” This fluid movement among groups suggests low social anxiety, high group cohesion, and a psychologically safe classroom environment where students feel welcomed and at ease across peer clusters. Teachers consistently noted that these relational and autonomy-supportive practices generated a ripple effect of social warmth. Students became more comfortable, less pressured to perform for validation, and more included, supporting one another in a more inclusive environment. Allan aptly described this shift: “They didn't have to prove anything… as long as they met the requirements and felt that they were doing good.”

Such reflections highlight how agency—when grounded in cultural values and supported by effective classroom design—empowers students to belong, connect, and flourish without fear of judgment. Research indicates that when students feel a sense of autonomy, competence, and connection with others, they tend to feel more energized and engaged (Vergara-Torres et al., 2021). This claim is further supported by studies emphasizing that when teachers adopt autonomy-supportive practices and help meet students' psychological needs, it boosts students' vitality and contributes to their overall wellbeing (Campoamor-Olegario et al., 2025; Candeias et al., 2024). Taken together, these findings reinforce the value of providing students with meaningful choice and agency in PE classes as a pathway to both personal and social flourishing.

#### 3.1.4 Theme summary: reclaiming human connection through relational pedagogy

The theme of creating meaningful connections illustrates how the PERMAH framework served not only as a pedagogical tool but also as a relational bridge, reconnecting teachers, and students after the isolating experience of remote learning. By re-establishing meaningful human connections, PERMAH helped restore the interpersonal dimensions of education that are essential for wellbeing, engagement, and holistic development.

In this study, PERMAH amplified deeply rooted Filipino cultural values. The teachers created a learning environment where autonomy and belonging could coexist by honoring *loób* (inner self) and *kapwa* (shared identity). As a result, PERMAH-infused PE classes fostered students' academic, physical, emotional, and relational growth in ways that felt authentic to their inner selves and attuned to others. These findings align with existing research on positive education frameworks. Seligman's (2011) model emphasizes that wellbeing and positive relationships enhance learning outcomes. Studies have consistently shown that supportive student-teacher relationships can significantly improve academic performance and emotional health (Kiuru et al., 2020; Zhang, 2024; Zheng, 2022). Fredrickson (2009) further supports this by highlighting how social connectedness contributes to emotional wellbeing and cognitive functioning—key elements in the learning process.

In the PERMAH-infused classes observed in this study, both students and teachers experienced a deepened sense of community and collaboration that extended beyond the classroom. This finding aligns with educational research that emphasizes the importance of relational pedagogy and supportive relationships in enriching the learning experience (Havik and Westergård, 2020; Li et al., 2022; Manchanda et al., 2023).

### 3.2 Nurturing safe spaces

This second primary theme reflects how the PERMAH framework fostered physical, emotional, and psychological safety within the PE learning environment. Teachers shared how their classrooms had transformed into inclusive and welcoming spaces where students felt freer to express themselves, take risks, and engage without fear of failure or judgment.

A key observation was the student's positive response to the shift from performance-based expectations to a focus on personal growth. This pedagogical change created space for deeper forms of connection, particularly through the cultural dynamic of *pakikipagpalagayang-loób* (mutual understanding and emotional attunement achieved through sincere interaction), which “occurs when people feel secure about revealing their sentiments and thoughts, are not ashamed of their actions, and show unconditional trust” (Santiago and Enriquez, 1976, as cited in Aguiling, 2024, p. 4).

Honoring *loób* allowed teachers to cultivate classrooms where students felt psychologically safe and emotionally seen. In PERMAH-infused PE classes, this honoring of loob facilitated *pakikpagpalagayang*-*loób* to emerge organically, reinforcing trust and empathy as the foundation of classroom interactions. This culturally grounded expression of mutual understanding reflects the concept of empathy, an essential relational skill that global educational programs are increasingly aiming to cultivate (Cuff et al., 2014). Thus, PERMAH-supported safe spaces are not only aligned with Filipino cultural values but also with broader global goals of relational and inclusive education.

#### 3.2.1 Reframing learning through emotional and psychological safety

A central element in nurturing safe spaces in PERMAH-infused PE classes was the deliberate shift in pedagogical mindset—from a focus on performance-driven outcomes to one on personal growth and development. This reorientation prioritized individual wellbeing over comparison, reducing stress, and increasing self-awareness. Allan encapsulated this transformation by expressing a desire to move “from the fitness objective to the wellness objective.” He proposed developing distinct courses such as “walking for fitness” vs. “walking for wellness.”

This focus on internal growth was consistently observed across PERMAH classes. Students were encouraged to evaluate their own progress rather than comparing themselves to others. Allan recounted one of his students' reflections, “I am competing with myself rather than competing with somebody else, and I'm happy where I am… I know I did well, and I am happy with that.”

Such statements reflect a reduction in anxiety and an increase in intrinsic motivation, made possible by a psychologically safe environment that fosters self-directed learning. Shean and Mander (2020) affirm that emotional safety, built through respectful relationships, empathic communication, and clear boundaries, is essential to a positive learning environment, supporting students' wellbeing, identity development, and academic success. This assertion is echoed by more recent studies showing that when students feel psychologically safe, protected from negative repercussions and judgment, they are more likely to express themselves authentically, build stronger connections, and engage more deeply in learning; such environments also reduce stress and anxiety while enhancing self-efficacy (Fisher and Rosikiewicz, 2025; Kwon et al., 2020; Schwarz et al., 2025).

This shift also gave rise to *pakiramdam—*a culturally embedded sensitivity or intuitive awareness that allows individuals to “feel with” others and respond with empathy, compassion, and care (Dayson et al., 2024). Reduced competitiveness and heightened self-awareness fostered deeper attunement not only to the self but also to others, reinforcing emotionally intelligent and empathetic peer relationships.

Moreover, the PERMAH environment empowered students to take ownership of their learning. Dahlia observed that students appeared less preoccupied with simply fulfilling course requirements. One student, for example, articulated a broader shift in priorities, saying she was more focused on “my growth, on my learning, on developing my relationship, and how I can grow with other people.” This orientation toward relational and emotional development also reflects deeply rooted Filipino values such as *loób* and *pagpapakatao*.

These cultural values align with global calls for whole-person education (Inter-Agency Network for Education in Emergencies, 2019) and character-based approaches to learning, which emphasize moral and emotional development as central to quality teaching (Kristjánsson et al., 2024). In this light, the PERMAH framework not only enhanced emotional and psychological safety but also served as a culturally grounded pathway to more humane, student-centered education.

#### 3.2.2 Enabling authentic expression

Participants highlighted the emotional openness and comfort that emerged in PERMAH-infused classes. These learning environments became sites where students felt safe to express their thoughts and emotions authentically. Brenda observed a “more natural flow of emotion” among students in her PERMAH class, which she attributed to activities intentionally designed to foster a safe and accepting atmosphere: “(The activities) made them feel comfortable… made them feel open to express more of what they feel.” Such emotionally engaging practices normalized expression and helped create a climate of psychological safety that extended to peer interactions.

This openness expanded even to topics typically considered sensitive or private. Fred shared that students in his class felt secure in discussing romantic interests or personal struggles: “It gave them courage to express unspoken feelings.” The classroom had become a relational space where *pakikipagpalagayang-loób—*the easing in of inner selves—could take root. In this trusting environment, students felt seen, heard, and understood, not only by their teachers but by one another.

Dahlia echoed this sentiment, describing the PERMAH class atmosphere as “a comfortable group… [you] know that you belong... and that you're welcome.” This sense of belonging stood in contrast to the more performance-oriented tone of her non-PERMAH class. While both groups showed technical proficiency, the teacher noted that students in the PERMAH group appeared to express a deeper emotional connection to their movements: “It translated very well with the movements... as compared to the non-PERMAH group, wherein they're more focused on the techniques.”

Together, these insights reveal that emotional safety and emotional expression were not accidental byproducts but rather intentional outcomes of the PERMAH-infused learning environment. As Gardner and Prasad (2022) and Leroy et al. (2021) point out, when students feel safe enough to share their honest thoughts and feelings, especially when encouraged to consider others' perspectives, it leads to stronger group relationships, deeper understanding, and better collaboration. Rooted in cultural values and relational pedagogy, these spaces enabled students to bring their whole selves into the classroom, fostering not just skill development but also emotional depth and human connection.

#### 3.2.3 Strengthening community through relational bonding

A defining feature of a nurturing learning environment is its ability to foster a sense of community that extends beyond the classroom. In PERMAH-infused PE classes, students, and teachers built connections that transcended formal instruction, reflecting a deeper relational culture. Brenda recalled how her class developed a warm rapport with the Tennis Court security guard: “Sometimes, we would invite him to eat with us, and there were a lot of things being shared over breakfast.” Such moments reflect *kapwa* in action—a shared identity grounded in dignity, mutual regard, and inclusivity (Enriquez, 1986).

Informal class gatherings like these create space for meaningful conversations where the teacher and students can share their personal experiences and thoughts without fear of judgment. This emotional openness reflected a healthy classroom climate shaped by *pakikipagpalagayang-loób* and *pakikiisa* (Enriquez, 1986). Gabby noted that these connections often carried on well after class: “Normally, the discussions and sharing continue even after my classes. And I noticed that the interactions are richer in terms of frequency of exchanges, and there's more humor and jokes shared, and sometimes, non-aikido-related conversations.”

Such ongoing, lighthearted conversations suggest that emotional bonding and psychological safety extend beyond formal instructional time. Teachers and students began to relate to each other not just as roles within a class but as fellow human beings. As Ivan noted, students even organized “food trips,” while Hilda recalled students showing up to support each other's performances, bringing warmth, care, and solidarity into shared spaces.

This belief in shared humanity fostered a collective responsibility to care for one another. Busiol and Lee (2015) corroborate that strengthening classroom relationships through trust and peer support networks is not only culturally resonant but also essential for building student resilience and fostering collective wellbeing. In education, such relational bonding is essential, as student wellbeing is closely linked to empathy, a sense of belonging, and the feeling of being valued, not just within the classroom walls but also in the broader learning community. Through PERMAH, students did not just experience classroom harmony—they experienced genuine community.

#### 3.2.4 Theme summary: nurturing safe spaces for emotional openness and belonging

In summary, the PERMAH framework facilitated the creation of emotionally grounded, inclusive, and affirming environments where students could be vulnerable, authentic, and connected. These safe spaces were characterized by positive emotions, reduced stress, non-competitive mindsets, increased emotional expression, and a strong sense of group belonging. Together, these elements enable students to re-engage with physical activity and social interaction in the post-pandemic classroom with confidence and joy.

A key relational value in these environments was *pakiramdam*—a culturally rooted sensitivity to and concern for the feelings of others (Pe-Pua and Protacio-Marcelino, 2000). Pakiramdam nurtured the emotional attunement and empathy necessary for creating spaces of psychological safety where both students and teachers could share openly and relate meaningfully.

These findings align with existing research, which indicates that a supportive and inclusive environment enhances students' academic engagement and personal development (Anokam and Ipem, 2022; **?**). By reducing stress and competition, the PERMAH model enabled students to focus on self-improvement and relational growth—key drivers of sustained motivation (Dorri Sedeh and Aghaei, 2024).

The value of mutual respect, shared humanity, and co-construction of learning environments rooted in care and dignity aligns with relational pedagogy (Su and Wood, 2023). Rooted in Filipino psychology and informed by global educational theory, PERMAH helped build spaces where students and teachers could grow, not in isolation, but through a shared sense of kinship.

### 3.3 Cultivating enjoyment and engagement

This third primary theme centers on how PERMAH-infused PE classes fostered a dynamic learning atmosphere where students experienced joy, energy, and meaningful engagement. Distinct from the earlier themes of connection and safety, this theme emphasizes the active participation and intrinsic enjoyment that emerged when students were not only comfortable but also genuinely invested in their learning experiences.

Teachers consistently observed heightened enthusiasm, sustained participation, and greater emotional investment in physical activities. Student's demonstrated regular attendance, delivered richer outputs, and expressed deeper satisfaction with their performance. What surfaced was not just compliance or comfort, but delights in movement, play, and shared discovery.

This heightened engagement was anchored in three culturally resonant drivers: clear goals *(layunin)*, opportunities for creative exploration (*malikhain)*, and the shared joy of learning together *(katuwaan)*. These elements nurtured both individual motivation and collective momentum. For instance, teachers noted that students displayed excitement in preparing routines, took initiative in class activities, and often extended their efforts beyond required outputs, reflecting a shift from passive participation to self-directed enthusiasm.

Kern (2022) explains that engagement involves three interrelated facets: behavioral (participation and adherence to class expectations), emotional (interest, enjoyment, and a sense of belonging), and cognitive (focused attention, use of strategies, and emotional regulation). The PERMAH strategies supported all three dimensions. Students were not only physically present but also emotionally engaged and mentally attuned—key markers of authentic learning.

In this way, PERMAH did more than create a supportive classroom—it made PE a space for pleasure, passion, and purposeful activity. The classes became venues where students flourished through movement, creativity, and collective enjoyment, redefining PE as a deeply fulfilling, whole-person experience.

#### 3.3.1 Promoting consistent participation and purposeful goal-setting

One of the most visible indicators of heightened engagement in PERMAH-infused PE classes was the improvement in attendance and retention. Allan noted a “lower number of drops in the PERMAH-infused class,” suggesting that students were more inclined to remain enrolled and participate consistently. Similarly, Carla observed that there were “more attendees” and greater enthusiasm around submitting course outputs: “They're excited to share.” These behavioral markers point to more than just enjoyment—they signal genuine motivation and emotional investment in the learning process.

This enthusiasm was rooted in *pakikiisa*, a Filipino value of collective unity and participation. When students feel supported, valued, and part of a learning community, their participation becomes more than a task—it becomes a shared experience. *Pakikiisa* captures this sense of belonging and cooperative engagement that energized PERMAH classrooms.

Enjoyment was also grounded in personal relevance and purposeful goal-setting. Mila noted that students “had a sense of purpose” when they were encouraged to set their fitness or performance goals early in the semester. This intentional process gave PE a more meaningful and personalized dimension: “PE allowed them to express their personal goals... they learned to set them at the beginning of the semester, and they monitored themselves.”

Such goal-setting fostered autonomy, ownership, and self-direction—key ingredients for sustained engagement. Research highlights that when students set clear, meaningful goals, they are more likely to stay focused, monitor their progress, and remain engaged, contributing to both academic success and lasting motivation (Bruhn et al. (2017) play a key role in this process by weaving goal-setting into their daily practice (Baghurst et al., 2015; Criss et al., 2024). These efforts are even more effective when supported by clear benchmarks and timely feedback, helping students stay on track and develop stronger self-regulation skills (Hauser, 2018).

By anchoring participation in *layunin* (purpose), the PERMAH framework transformed PE from a compliance-driven subject to a platform for self-expression, growth, and relational commitment. Students were not just showing up—they were showing up with intention. In this way, PERMAH supported not only physical activity but also the deeper psychological and cultural dimensions that make learning personally and socially meaningful.

#### 3.3.2 Fostering creativity, fun, and intrinsic motivation

PERMAH-infused PE classes not only sparked stronger participation but also yielded more imaginative and higher-quality student outputs. Teachers observed a noticeable increase in creativity, with Brenda describing a student's work as “a more creative way of interpreting the dance.” Hilda similarly remarked that students were more expressive in performance because “they're more motivated to engage themselves in the activity.” The infusion of positive emotions and meaningful relationships fostered a classroom culture where students felt free to explore, innovate, and take ownership of their learning. Positive experiences in PE, such as overcoming challenges and building a healthy body image, can boost students' self-confidence, strengthen social connections, and support overall well-being (Rojo-Ramos et al., 2025).

This form of creative engagement reflects the Filipino value of *malikhain*—a deeply personal, intuitive expression of creativity that originates from *loób* (inner self). When students feel emotionally safe and socially supported, they are more willing to take risks, express original ideas, and take pride in their growth. Apparently, PERMAH created the conditions for this creative confidence to surface. Research reinforces that when learning environments cultivate intrinsic motivation; students tend to exhibit greater creativity and sustained engagement (Saito et al., 2025; Zhang et al., 2021).

Playfulness and humor played a central role in boosting intrinsic motivation. Jason recalled that during practical exams, the PERMAH group was marked by “a lot of cheering, involvement, and joking around,” cultivating a lighter atmosphere that reduced pressure and built camaraderie. Significantly, this fun-filled environment did not compromise academic performance. On the contrary, Fred noted that “the top performer was from the PERMAH class,” while Ella reported better outcomes in her PERMAH walking-for-fitness class, even though students appeared to be having more fun.

Allan affirmed these observations with concrete results: “They are walking faster. They are fitter, and they know that... They are very, very happy with how they perform.” This internalized recognition of progress, paired with enjoyment, signals the development of intrinsic motivation. Students were not merely performing for grades or approval; they were invested in their improvement and wellbeing.

The fusion of *katuwaan* (shared joy) and performance challenges the notion that rigor and fun are mutually exclusive in classroom learning. In PERMAH-infused classes, emotional investment and high-quality outcomes coexisted, suggesting that when students are supported, engaged, and genuinely enjoying themselves, excellence often follows.

#### 3.3.3 Re-energizing teacher practice and learning culture

The positive energy generated in PERMAH-infused PE classes was not limited to students—it also invigorated teachers. Witnessing their students' joy, creativity, and meaningful participation inspired educators to reflect more deeply on their practices and renew their commitment to engaging, learner-centered teaching. Jason expressed a renewed desire to expand his strategies, saying he wanted to “have more activities to engage more of my students,” indicating both renewed motivation and pedagogical momentum.

This sense of renewal went beyond enthusiasm. For some teachers, it sparked deeper reflection and intentional innovation. Gabby shared that he began rethinking his teaching approach, suggesting a shift toward more responsive and reflective pedagogy. Brenda described this transformation as a creative awakening: “My creativity was fortified... as a teacher in dealing with the lesson, which I really enjoyed.” Her experience illustrates how the emotional and cognitive energy flowing through the class also revitalized her role as a teacher. Research confirms that this kind of re-energizing is vital to building a dynamic and engaging culture, primarily when supported by professional development that encourages reflection and culturally responsive practice (McChesney and Cross, 2023; Tresserras and Sangrà, 2023).

The re-energizing effect of PERMAH reflects *malikhain*, not only as a student value but also as a driver of teacher creativity. As students began to thrive with joy, purpose, and ownership, their teachers were inspired to meet that energy with greater intentionality, creativity, and care, fostering a mutually reinforcing cycle of engagement, innovation, and shared flourishing. These interconnected experiences of purpose, motivation, and renewal resonate with findings that underscore the importance of integrating goal-setting, cultivating intrinsic motivation, and revitalizing teacher practices to sustain joyful and engaged learning. When students have clear goals and opportunities for creative, meaningful participation, and when teachers feel recharged and reflective, engagement deepens in the classroom (Baghurst et al., 2015; Zhang et al., 2021).

#### 3.3.4 Theme summary: students' joyful purpose and sustained engagement

In summary, the teachers' accounts suggest that the PERMAH framework may have contributed to the creation of a PE environment where students looked forward to class, participated fully, and derived deep personal meaning in their class engagement. Far from being driven by compliance or competition, students showed motivation rooted in purpose *(layunin)*, expressed themselves through creative expression *(malikhain)*, and participated in activities with shared joy *(katuwaan)*. Together, these elements appeared to have led to a profound sense of *ginhawa*—a state of wellbeing marked by ease, enjoyment, and fulfillment.

Importantly, this enjoyment was not shallow or merely recreational. It was deeply connected to students' goals, creativity, relationships, and self-development. The PERMAH approach demonstrated that fun and rigor are not mutually exclusive, but appear to be mutually reinforcing—when students feel joy and ownership in what they do, they are more likely to persist, excel, and grow.

These findings affirm Braun and Clarke (2006) self-determination theory, which emphasizes that intrinsic motivation arises when individuals engage in activities for the inherent satisfaction they provide rather than for external rewards or recognition. In this study, the teachers consistently observed that PERMAH strategies played a role in supporting intrinsic motivation, where students tended to be more eager to participate, take pride in their progress, and report a stronger sense of accomplishment. This outcome also resonates with the work of Lawrie and Mendoza (2024), who argue that positive psychology frameworks can enhance engagement and enjoyment in educational settings. By emphasizing personal growth and shared success over competition, the PERMAH model cultivated a classroom culture where students were emotionally invested, socially connected, and cognitively engaged. Ultimately, this theme underscores the importance of creating learning environments where joy, meaning, and achievement coexist (Fredrickson, 2009). In PERMAH-infused classes, student engagement was not only sustained—it was energized by purpose, creativity, and a sense of community.

### 3.4 Enhancing teaching fulfillment

This fourth primary theme highlights how PERMAH-infused PE classes positively impacted not only student experiences but also the inner lives and professional fulfillment of teachers. Instructors described a renewed sense of purpose, deeper satisfaction in their work, and a stronger emotional connection to their students, indicating that PERMAH fosters a more humanizing and life-affirming teaching experience.

Unlike previous themes centered on student engagement or classroom dynamics, this theme underscores the internal transformation of the teacher. The PERMAH approach encouraged educators to reconnect with their *pagkatao*—the wholeness of one's personhood—and to experience teaching not merely as a duty, but as a meaningful and relational vocation. For these teachers, the classroom became a space for *pakikipagkapwa—*a shared recognition of dignity and interconnectedness—where each interaction was an opportunity to live out one's values and deepen their sense of self (Javier, 2017).

PERMAH-inspired pedagogy reignited joy and creativity in the teaching process. Teachers spoke of being more reflective, intentional, and emotionally invested in their teaching. They reported finding renewed purpose in their roles and experiencing alignment between their work and personal values. These findings are consistent with Wammerl and Lichtinger's (2025) study, which found that teachers who participated in the training program that helped them implement PERMA in the classroom, enhanced their wellbeing and fostered an emotionally supportive environment. In sum, this theme illustrates that PERMAH revitalizes the educators who lead learning, supporting their flourishing alongside that of their students.

#### 3.4.1 Rediscovering joy and purpose in teaching

Teachers frequently expressed that teaching became more enjoyable, meaningful, and personally rewarding in the PERMAH-infused PE classes. Allan reflected, “I really enjoyed teaching this PERMAH class,” noting that it was “more fulfilling as an instructor” due to the increased interaction and positive outcomes. Similarly, Brenda shared that the PERMAH class “fortified [her] creativity” and enabled her to enjoy the lesson planning and delivery more, as evidenced by her statement, “I really enjoyed it.”

These accounts reflect findings in current literature, which show that teachers who experience wellbeing across the PERMA domains report greater job satisfaction (Dreer, 2021), reduced workplace stress and stronger resilience (Fitzsimons et al., 2025), as well as enhanced teaching effectiveness and overall life satisfaction (Qin et al., 2024; Wammerl and Lichtinger, 2025). Brenda's commitment to continue using the PERMAH approach—“to do it with the rest of my class for as long as I can teach” —mirrors this evidence, suggesting that teaching in a PERMAH context not only boosts joy but also reinforces long-term professional dedication. This account supports Wammerl and Lichtinger's (2025) findings that teachers in PERMAH-based programs reported higher engagement, deeper flow experiences, and greater use of their character strengths in school.

Despite the additional effort required to design PERMAH-infused curricula, teachers described the experience as rejuvenating. Ella noted, “I noticed that I was eager to attend the PERMAH sessions,” revealing a renewed motivation and emotional energy for teaching. Hilda echoed this, saying she felt “freer” while teaching the PERMAH class. This shift enabled more authentic interactions, allowing her to express herself more personally and professionally.

These reflections illustrate how the PERMAH framework not only benefits students but also supports the flourishing of teachers, restoring a sense of joy, purpose, and vitality in their practice.

#### 3.4.2 Building compassionate and responsive teacher-student relationships

Applying PERMAH encouraged teachers to connect with students on a more personal level—beyond academic instruction—fostering deeper trust, care, and emotional openness. Brenda shared how the experience led her to connect with students: “It drew me a lot closer to some of the students who were able to open up to me,” even forming regular “buddy talks” with three students. These relational moments enriched her teaching experience and contributed to a classroom culture rooted in empathy and mutual respect.

PERMAH also prompted teachers to become more reflective and intentional in their practice. Fred explained, “You just have to adjust for them… because we're aware that it's the PERMAH group,” indicating a heightened sensitivity to students' emotional and developmental needs. For some, this meant softening their tone, becoming friendlier, or finding a balance between structure and compassion, thereby fostering an atmosphere where students felt seen, heard, and supported. The study of Candeias et al. (2024) has also shown that when teacher develop greater self-awareness and practice self-regulation, they are better able to respond to their students with empathy and care, creating a classroom where everyone feels safe, supported, and emotionally connected.

This shift in teacher–student dynamics reflects not only the principles of positive psychology but also resonates with core values in Filipino Psychology. The move toward a more humanizing and relational pedagogy is deeply aligned with *pakikipagkapwa* (shared personhood), which calls for teachers to treat students with dignity and care as equals.

Teaching with compassion and integrity also reflects *paninindigan*—the conviction to stand firm in one's values and responsibilities. In Filipino culture, this means not merely stating a promise, but *pinangatawanan*—living it out through consistent action. As Javier (2017) emphasizes, teachers bring their values to life through their daily presence, care, and actions. Through this lens, PERMAH becomes more than a pedagogical model; it becomes a moral and cultural commitment.

Ultimately, this theme shows that PERMAH not only strengthens classroom relationships—it reaffirms teaching as an ethical and relational vocation. Compassionate teaching is an expression of *paninindigan*, a lived commitment to uphold dignity, empathy, and cultural rootedness in every interaction.

#### 3.4.3 Teaching rooted in Filipino values and shared humanity

Several teachers shared that the PERMAH approach brought out a more human side of teaching, helping them to reconnect with what it means to be truly human—*pagpapakatao* in Philippine culture. Hilda expressed, “I felt more human in my PERMAH class,” while Linda described the experience as “I also felt it more …that I was truly human,” signaling a shift from rigid instructional roles to a more empathetic, responsive teaching identity. These reflections mark a significant shift from rigid instructional roles to a “more human” approach to teaching, reflecting a deeper alignment between their personal values and teaching practices.

This sense of “being more human” suggests a deeper integration of personal values into teaching practice. It reframes teaching not merely as a series of tasks, but as an expression of *pagkatato*—one's core personhood. PERMAH served as a conduit through which teachers could live out their humanity in the classroom, reinforcing the idea that teaching is not just about what one does, but who one becomes in relationship with others. As Agbayani et al. (2022) observed, Filipino teaching is profoundly shaped by values like collectivism, social acceptance, and spirituality, which guide how educators build relationships and derive meaning from their work. This perspective is also reflected in the Department of Education's Indigenous Peoples' Education (IPEd) program, which promotes culturally responsive teaching grounded in respect, connectedness, personal relevance, and authenticity (Caingcoy et al., 2022). These principles are closely aligned with the PERMAH approach.

For Filipinos, a sense of belonging is intimately linked to finding *saysay*—or meaning—in life, which in turn nurtures wellbeing (Wapano and Paguta, 2022). Research illustrates that culturally relevant teaching and social justice education help teachers connect more effectively with diverse learners, an approach that corresponds with PERMAH's focus on empathy, relevance, and authentic relationships (Shiver et al., 2020; Vasquez et al., 2022). A person's deep sense of being human (*pagkatao*) is shaped not only by their relationships with others but also by a clear understanding of life purpose (Campoamor-Olegario et al., 2025). In this context, teaching grounded in *saysay* becomes a pathway to *ginhawa*—a culturally rooted state of wellbeing marked by happiness, fulfillment, and relief from life's burdens (Samaco-Zamora and Fernandez, 2016).

Through the PERMAH lens, teachers not only fostered student learning but also reconnected with their own humanity. Teaching became an act of *pakikipagkapwa, paninindigan*, and *pagpapakatao*—a lived embodiment of shared dignity, purpose, and wellbeing. This view resonates with the humanizing approaches to education, which emphasize empathy, cultural awareness, and social responsibility. In Physical Education, integrating culturally oriented activities can promote inclusivity and foster deeper cultural understanding, preparing students to become more socially aware and globally responsible (Pacadaljen, 2024).

#### 3.4.4 Theme summary: reclaiming purpose through co-flourishing

Ultimately, teachers saw themselves not only as facilitators but also as co-beneficiaries of the PERMAH approach. As Allan observed, “All the activities and the additional drills were beneficial to everyone, including the instructor and me.” This sentiment reflects a broader insight: PERMAH did not just enhance student outcomes; it also helped teachers reconnect with their sense of purpose, spark their creativity, and renew their emotional connection to teaching.

The theme of *enhancing teaching fulfillment* illustrates how PERMAH transformed PE instructors into more inspired, relational, and values-driven educators. Their testimonies affirm that when classrooms become spaces of *kaginhawaan* (wellbeing) and *pakikipagkapwa* (authentic connection and shared humanity), the positive impact extends not only to students but also to the educators themselves. In these spaces, teaching becomes not just a profession but a deeply humanizing experience.

These findings support prior research indicating that teaching practices focused on student wellbeing and engagement lead to higher levels of teacher satisfaction (Burić et al., 2024; Wang et al., 2024). Teachers in this study reported increased creativity, a more profound sense of fulfillment, and stronger emotional bonds with their students, echoing Turner and Thielking's (2019) observation that integrating positive education principles supports professional wellbeing. This is further supported by studies showing that teachers who cultivate supportive environments report higher professional satisfaction, lower stress levels, and increased self-efficacy (Jiang et al., 2024). Additionally, fostering a supportive and engaging learning environment, characterized by precise instructions, metacognitive strategies, and meaningful assessment, has been found to enhance both student learning outcomes and teacher satisfaction (Chowdhary, 2025).

At its core, this theme reveals that when teaching is grounded in *pakikipagkapwa*—a shared humanity rooted in mutual respect and empathy—it becomes fertile ground for *saysay* (meaning-making) and the unfolding of *pagkatao* (personhood), not only for students but also for teachers. PERMAH, then, is not simply a pedagogical framework; it is a pathway for co-flourishing—where both learners and teachers grow, thrive, and connect with the heart of education: to become more human, together. This theme resonates with the work of Cherkowski et al. (2018) and Wolbert et al. (2015), which emphasize that co-flourishing in education emerges when both teachers and students engage in a holistic learning experience grounded in wellbeing, shared purpose, and mutual growth. Recent research also confirms that PERMAH promotes teacher wellbeing and fosters a favorable, emotionally supportive school climate, highlighting its potential as a catalyst for systemic educational renewal (Wammerl and Lichtinger, 2025).

### 3.5 Thematic synthesis and general discussion

Integrating the PERMAH framework in PE classes played a role in influencing both students and teachers in profound and multifaceted ways. The themes—creating meaningful connections, nurturing safe spaces, cultivating enjoyment and engagement, and enhancing teaching fulfillment—reflect a shift from performance-driven instruction to a more holistic, relational, and wellbeing-oriented educational experience.

#### 3.5.1 PERMAH as a culturally resonant framework for wellbeing in PE

The teachers' narratives suggest that PERMAH not only functioned as a positive psychology model but also resonated deeply with Filipino cultural values and ways of relating. Its application fostered interpersonal bonds, emotional safety, and student agency, as well as a reimagining of PE as a space not merely for fitness but for flourishing. Students were observed to be more motivated, collaborative, and expressive. These are qualities nurtured in a learning environment that prioritized trust, empathy, and shared growth over comparison or competition.

These relational shifts closely aligned with Filipino Indigenous psychological concepts such as *kapwa, loób*, and *pakikiramdam*, which shaped both classroom culture and teaching practice. Rather than impose a Westernized model, PERMAH seemed to amplify what was already culturally embedded in the Filipino approach to human interaction. Table 3 presents these values, their corresponding definitions, and sample teacher statements that exemplify how they were enacted in practice.

**Table 3:** Filipino indigenous values in PERMAH-infused education: definitions and teacher exemplars.

**Value**	**Definition**	**Exemplar statements**
*Kapwa*	Shared identity, recognizing the dignity and humanity of every person.	(Ella) “After our activity, we stayed in a lagoon and made a big circle to discuss our feelings during the walk.” (Brenda) “Students invited even the security guard to join meals.”
*Loób*	Inner self, the internal dimension encompassing thoughts, feelings, and will.	(Allan) “Students expressed that they were 'competing with themselves' and felt happy with their own progress.” (Brenda) “Activities helped students feel comfortable and open to expressing what they felt.”
*Pakiramdam*	Sensitivity or empathy toward others' feelings and internal states.	(Allan) “The ability to work with someone and accomplish something together made them feel emotionally connected.”
*Pakikipagpalagayang- loób*	Establishing mutual trust, understanding, and emotional attunement.	(Brenda) “Regular 'buddy talks' fostered trust and emotional openness between teacher and student.“ (Fred) “Students expressed difficult feelings due to the trusting classroom atmosphere.”
*Pakikiisa*	Being one with the group, characterized by unity in purpose and participation.	(Kristine) “The entire class attended the culminating activity, complete with tarpaulin and balloons.” (Jason) “Students were proud that their classmates learned to swim and supported each other through the process.”
*Pakikisangkot*	Active involvement and participation in a shared endeavor.	(Dahlia) “Students asked to stay longer in class to continue practicing together.” (Hilda) “They helped each other with notes and choreography, sharing in the class GC.”
*Pagpapakatao*	Becoming fully human, embodying dignity, care, and moral responsibility.	(Hilda) “I felt more human in my PERMAH class, more connected and authentic.” (Brenda) “Teaching with PERMAH helped me live out my values and care more deeply.”
*Ginhawa*	Well-being: a state of relief, contentment, or flourishing.	(Allan) “Students are walking faster, fitter, and very happy with how they perform.” (Fred) “PERMAH made classes more enjoyable and helped students express themselves better.”
*Layunin*	Purpose or intentionality in action.	(Mila) “Students set goals at the beginning of the semester and monitored their own progress.” (Allan) “The shift from fitness to wellness enabled students to focus on purpose, not just outcomes.”
*Malikhain*	Creativity rooted in self-expression.	(Brenda) “PERMAH infused creativity naturally—students generated interpretations without being told.” (Brenda) “Outputs were more creative because students felt safer to explore ideas.”
*Paninindigan*	Conviction or living one's values with integrity.	(Brenda) “I'll do PERMAH with the rest of my classes for as long as I can teach.” (Fred) “Teachers had to adjust and stand firm in values to sustain relational teaching.”
*Saysay*	Meaning or significance in life and actions.	(Allan) “The PERMAH class helped me rethink my teaching as 'walking for wellness' with meaning.” (Brenda) ”The experience reconnected me with the *saysay* of teaching and students with purpose.”

#### 3.5.2 Enhancing teacher fulfillment and humanizing practice

The PERMAH approach also contributed to revitalizing the professional and personal experiences of teachers. Many expressed a renewed sense of joy, creativity, and alignment with their vocation. Through PERMAH, teaching became more relational and intentional. Teachers shifted from being mere transmitters of knowledge to being companions in their students' journeys—modeling pakikipagkapwa through acts of care, trust, and authentic presence.

This transformation reflects the Filipino value of pagpapakatao—a culturally rooted sense of personhood cultivated through moral responsibility and relational integrity. As teachers reconnected with their pagkatao (wholeness of self), they also reported enhanced wellbeing and deeper satisfaction in their work.

These findings align with international research suggesting that teaching practices rooted in wellbeing and relational pedagogy lead to higher professional satisfaction, lower stress, and increased self-efficacy (e.g., Burić et al., 2024; Turner and Thielking, 2019; Jiang et al., 2024). In this context, PERMAH is not simply a pedagogical strategy but a pathway toward humanized education that uplifts both students and teachers.

#### 3.5.3 Summary of insights and response to research questions

Taken together, the four emergent themes provide a coherent narrative that addresses the study's three core research questions. While the study is limited in scope, its findings offer insights into how wellbeing can be meaningfully and culturally integrated into PE classrooms, especially during the transition from remote to in-person learning.

- The themes *creating meaningful connections, nurturing safe spaces*, and *cultivating enjoyment and engagement* illustrate how Filipino PE teachers applied the PERMAH domains—particularly Positive Emotion, Engagement, Relationships, and Meaning—in post-pandemic classrooms. These themes address Research Question 1, which explored how PERMAH was implemented in PE teaching practice.
- These same themes also highlight observable differences between PERMAH and non-PERMAH classes in terms of student motivation, participation, and social-emotional wellbeing, thereby addressing Research Question 2 on comparative classroom dynamics.
- Finally, the theme *enhancing teaching fulfillment* responds to Research Question 3, capturing how the PERMAH framework influenced teachers' professional identities, creativity, and sense of purpose.

Taken together, the findings suggest that a culturally grounded adaptation of the PERMAH framework holds promise for fostering mutual wellbeing—or co-flourishing in post-pandemic higher education. In the Filipino PE context, where *pakikipagkapwa, loób*, and *saysay* (meaning) are deeply held values, PERMAH offered both a language and a lived experience of what truly lies at the heart of education: human connection, shared growth, and the pursuit of a flourishing life together.

### 3.6 Implications for policy and practice

These findings provide practical insights that inform teacher education, curriculum development, institutional priorities, and efforts to support the well-being of educators in higher education.

#### 3.6.1 Teacher education

A critical takeaway from this study is the value of formally integrating the PERMAH framework to prepare future educators to cultivate wellbeing through supportive and values-driven classrooms. Coursework and practicum experiences can incorporate Filipino cultural values such as *kapwa, loób*, and *pagpapakatao*, reinforcing a relational approach to teaching and learning.

#### 3.6.2 Curriculum design

This study's findings strongly support the integration of the PERMAH model into higher education curricula, particularly in physical education and related fields. Research by Seligman (2011) and Dorri Sedeh and Aghaei (2024) underscores the positive outcomes associated with incorporating wellbeing models in educational settings.

#### 3.6.3 Institutional Priorities

This study advocates for a shift from competitive, grade-driven models toward learning environments that prioritize personal development and collaboration. Evidence suggests that when students focus on self-improvement rather than comparing themselves to their peers, they are more likely to experience greater intrinsic motivation, leading to enhanced performance and overall wellbeing (Deci and Ryan, 2000; Fredrickson, 2009).

#### 3.6.4 Teacher wellbeing and professional development

Findings from this study indicate that the PERMAH also strengthens teacher wellbeing by fostering creativity, connection, and purpose in teaching. Institutions should support this by offering professional development in positive, student-centered pedagogy. Culturally responsive approaches grounded in Filipino values can sustain both teacher fulfillment and student flourishing.

In summary, PERMAH served as a teaching framework and a pathway to meaningful and lasting personal growth and transformation. This approach aligns with global calls for culturally responsive teaching (Gay, 2018) and a whole-person approach to education (Inter-Agency Network for Education in Emergencies, 2019), demonstrating how Indigenous values, such as *kapwa* and *pagpapakatao*, can provide powerful insights that enrich educational practices worldwide.

## 4 Reflexivity

Reflexivity was a critical and continuous process throughout the study. As qualitative researchers, we recognized that our backgrounds, experiences, and assumptions inevitably shaped our interactions with participants, data collection, analysis, and interpretation (Berger, 2015). To ensure rigor and transparency, we engaged in regular reflexive dialogue to examine how our positionalities influenced the research process. As co-authors, we brought different but complementary perspectives to this study. We are Filipino educators based in the Global South, deeply committed to teacher development, student wellbeing, and culturally grounded educational approaches.

As an experienced educator and sport psychologist, the first author brought a deep understanding of psychosocial dynamics in educational settings. This expertise offered valuable insights into participant narratives, particularly those related to motivation, wellbeing, and teacher-student relationships. At the same time, the author remained mindful of the tendency to overemphasize psychological interpretations and actively sought input from the other team members to balance the analysis.

The second author, an associate professor of educational psychology, contributed her extensive background in socio-emotional learning, action research, and interdisciplinary work focused on PERMAH to teaching and curriculum. Her engagement with Filipino psychological concepts—*loób, kapwa*, and *pagpapakatao*—helped shape the culturally responsive and relational lens through which we approached the study and its analysis.

Notably, both authors were researchers and educators who experienced firsthand the challenges of remote teaching, reintegration, and emotional fatigue during the COVID-19 pandemic. These lived experiences fostered a heightened sensitivity to the emotional effort, pedagogical adjustments, and relational dynamics described by the participants. Our attempts to reconnect with students and reflections on teaching meaningfully during the crisis shaped our deep empathy for the PE instructors. These parallel journeys allowed us to listen attentively, code more intuitively, and interpret themes with grounded insight into the real-life complexities of post-pandemic education.

Working within the same public university system during the educational recovery period, we shared a strong sense of connection with the PE teachers who participated in the research. Their resilience, creativity, and care profoundly resonated with our efforts to support wellbeing. In many ways, the findings reflect the stories of those involved and our shared aspiration to reclaim joy, connection, and meaning in teaching after a disruption.

## 5 Limitations

### 5.1 Sample size and transferability

The study was conducted with a relatively small sample size from specific higher education courses. While the findings provide valuable insights, the results are highly contextual to this demographic; as such, they may not be transferable to all educational contexts. Future research with larger, more diverse samples—for instance, from K-12 or non-urban schools—would enhance the robustness of the findings and enable a broader generalization of the results.

### 5.2 Short-term nature of the study

The study was conducted over a single semester. Although the positive effects of the PERMAH model were evident, it remains unclear whether these benefits will persist over the long term. Longitudinal studies would provide more comprehensive insights into the sustainability of these outcomes and the long-term impact of the PERMAH model on both students and teachers.

### 5.3 Teacher bias

Since this study relied on self-reported data from teachers, there is a risk of response bias. Participants may have been inclined to present more favorable outcomes due to their personal investment in the PERMAH model. Additionally, while the research focused on the perceptions and practices of Filipino PE teachers, the exclusion of student voices limits the ability to triangulate findings and fully assess the effectiveness of PERMAH integration on student engagement, wellbeing, and learning outcomes.

The teachers also encountered several practical challenges during the implementation. Many noted that online reflection sessions made it difficult to foster genuine interaction, with some participants disengaging. Implementing PERMAH also required significantly more effort than traditional classes because they had to infuse PERMAH into their classes intentionally.

Rain, high temperatures, holidays, and reading breaks disrupted schedules and sometimes led to the cancellation or rescheduling of planned activities. Some teachers found it challenging to maintain a clear boundary between their PERMAH and non-PERMAH classes, particularly when teaching the same lesson back-to-back.

They also reported that there were instances where certain activities, such as daily emotional check-ins, began to feel repetitive. Teachers had to modify these routines creatively to keep students engaged and responsive.

Future studies could address these challenges by incorporating student perspectives, utilizing classroom observations, and collecting additional data on student outcomes and wellbeing. Longer-term studies and more diverse educational settings would also help assess the sustainability and broader applicability of PERMAH-informed teaching across various contexts.

Future studies may build on these findings by incorporating student perspectives, gathering student reflections, conducting classroom observations, or collecting wellbeing survey data to gain a deeper understanding of how PERMAH-informed practices influence classroom dynamics from multiple vantage points. Comparative studies across different institutions or educational levels may also shed light on how PERMAH is adapted in varied contexts and which elements resonate most strongly within specific cultural or academic settings.

## 6 Conclusion

This study aimed to investigate the impact of the PERMAH model on students and teachers in higher education, with a focus on student engagement, teacher fulfillment, and overall learning outcomes. Through an in-depth examination of the lived experiences of PE teachers during the transition from remote to in-person learning, the study successfully addressed its research questions by uncovering how each domain of the PERMAH model was implemented in practice and how these practices influenced both student and teacher experiences.

The findings suggest that, based on teachers' reflections, the PERMAH-infused approach contributed to fostering student engagement by promoting meaningful connections, emotional safety, and intrinsic motivation, which manifested in improved attendance, increased enthusiasm for participation, and deeper learning connections. These behavioral and emotional indicators of engagement affirm the models' effectiveness in supporting post-pandemic re-engagement. Furthermore, the study indicates that PERMAH-informed practices may enhance teacher fulfillment by enabling more values-aligned and relational approaches to teaching. Teachers described experiencing renewed purpose, relational depth, and professional growth—outcomes that closely align with Filipino Indigenous concepts such as *pakikiramdam, pakikipagkapwa*, and *pagpapakatao*.

In achieving its objectives, the study provides empirical evidence that PERMAH can be more than a universal framework—it can also be culturally responsive and transformative through local psychological and sociocultural lenses. The research highlights how Filipino PE teachers enacted PERMAH not as a prescriptive model but as a flexible guide to draw from cultural values toward holistic wellbeing. This underscores how universal conceptions of wellbeing can be localized to meet the unique cultural and educational realities of the Global South.

By capturing how Filipino educators utilized PERMAH to address both the lingering impacts of pandemic-related disruptions and longstanding systemic constraints, this study provides a valuable perspective on ongoing conversations about decolonizing the concept of positive education as defined in Global North contexts. It affirms that PE settings are powerful platforms for advancing the wellbeing of both students and teachers, particularly when approaches are culturally informed and relationally grounded. As such, this study not only answers its central questions but also opens new pathways for reimagining education as a site of recovery, resilience, and flourishing.

## Data Availability

The original contributions presented in the study are included in the article/supplementary material, further inquiries can be directed to the corresponding author.
